# 1,3-Butadiene: a ubiquitous environmental mutagen and its associations with diseases

**DOI:** 10.1186/s41021-021-00233-y

**Published:** 2022-01-10

**Authors:** Wan-Qi Chen, Xin-Yu Zhang

**Affiliations:** School of Public Health, Hongqiao International Institute of Medicine, Shanghai Jiao Tong University School of Medicine, Shanghai, 200025 China

**Keywords:** Butadiene, Environmental mutagen and carcinogen, Human exposure, Microenvironments, Urinary biomarkers, Cigarette smoking, Lung and larynx cancers, Adverse health effects, Children’s health

## Abstract

1,3-Butadiene (BD) is a petrochemical manufactured in high volumes. It is a human carcinogen and can induce lymphohematopoietic cancers, particularly leukemia, in occupationally-exposed workers. BD is an air pollutant with the major environmental sources being automobile exhaust and tobacco smoke. It is one of the major constituents and is considered the most carcinogenic compound in cigarette smoke. The BD concentrations in urban areas usually vary between 0.01 and 3.3 μg/m^3^ but can be significantly higher in some microenvironments. For BD exposure of the general population, microenvironments, particularly indoor microenvironments, are the primary determinant and environmental tobacco smoke is the main contributor. BD has high cancer risk and has been ranked the second or the third in the environmental pollutants monitored in most urban areas, with the cancer risks exceeding 10^-5^. Mutagenicity/carcinogenicity of BD is mediated by its genotoxic metabolites but the specific metabolite(s) responsible for the effects in humans have not been determined. BD can be bioactivated to yield three mutagenic epoxide metabolites by cytochrome P450 enzymes, or potentially be biotransformed into a mutagenic chlorohydrin by myeloperoxidase, a peroxidase almost specifically present in neutrophils and monocytes. Several urinary BD biomarkers have been developed, among which *N*-acetyl-*S*-(4-hydroxy-2-buten-1-yl)-L-cysteine is the most sensitive and is suitable for biomonitoring BD exposure in the general population. Exposure to BD has been associated with leukemia, cardiovascular disease, and possibly reproductive effects, and may be associated with several cancers, autism, and asthma in children. Collectively, BD is a ubiquitous pollutant that has been associated with a range of adverse health effects and diseases with children being a subpopulation with potentially greater susceptibility. Its adverse effects on human health may have been underestimated and more studies are needed.

## Introduction

1,3-Butadiene (BD), a colorless gas, is an important petrochemical manufactured in high volumes that is primarily used to produce synthetic rubber and thermoplastic resins. BD is a ubiquitously environmental pollutant because it is formed as a product of incomplete combustion of fossil fuels and biomass [1]. It is one of volatile organic compounds (VOCs) monitored routinely in the ambient air.

BD was determined to be a human carcinogen by the U.S. Environmental Protection Agency (EPA) in 2002 [1] and by the International Agency for Research on Cancer (IARC) in 2008 (Group 1 carcinogen) [2]. BD induces lymphohematopoietic cancers in occupationally-exposed workers [1, 2]. It is one of 187 hazardous air pollutants (HAPs), also known as air toxics, as defined by EPA [3].

BD was fully reviewed in the official document “Health Assessment of 1,3-Butadiene” issued by EPA in 2002 [1] and in Volume 97 of the IARC Monographs on the Evaluation of Carcinogenic Risks to Humans published in 2008 [2]. In the two documents, almost all aspects of BD were discussed in great detail. In 2012, IARC published Volume 100F and provided an update on BD based on newly available data [4]. In addition, the metabolism, DNA adducts, toxicology, genotoxicity, and the carcinogenic modes of action of BD were also reviewed in 2007 and 2010 [5–8]. However, since then many new studies, in particular, the epidemiological studies concerning the associations with several diseases in children, have been published but have not been reviewed yet. Thus, in this review, we will provide an update on major progress in BD-related studies over the last decade. However, we do not try to include all BD-related literature published during this period. Rather, we will focus on progress in the following aspects: environmental sources, airborne concentrations, human exposure, cancer risks, metabolism, urinary biomarkers, genotoxicity in humans, and associations with diseases. Additionally, because BD is one of the major constituents in tobacco smoke, we discuss the possible contribution of BD to tobacco smoking-associated diseases, with the focus being on cancers of lung and larynx.

### The environmental sources of BD

BD has many environmental sources, including industrial emissions from production of BD, rubber, and resins, automobile exhaust, tobacco smoke, and exhaust from biomass burning and cooking [1]. According to EPA, automobile exhaust and miscellaneous combustion sources contribute 78.8% and 19.6% of the total BD emissions in the environment, respectively. The industrial emissions account for only 1.6% of the total BD amounts in the environment, in spite of the fact that tremendous amounts of BD are produced and used in petrochemical and synthetic rubber industries [1].

Automobile exhaust is the primary source of BD in the environment due to the enormous amounts of fuels consumed by automobiles [1]. The average emission factor of vehicles was reported to be 2.1 ± 1.5 mg/km in 1997 [9] and 0.7 ± 0.4 mg/km in 2018 [10]. However, the emission factors are substantially greater in congested traffic compared with those under highway cruise conditions [11]. The BD emissions reduce when vehicles use fuels containing ethanol [12] but increase for biodiesel [13]. It has been estimated that on-road and non-road motor vehicles contribute to 51% and 20% of the BD emissions in urban counties in the United States, respectively [14].

Cigarette smoke contains significant amounts of BD and can thus be the primary source in indoor air [1]. The yields of BD in cigarette smoke are 12-92 μg/cigarette in the mainstream smoke [15–17] and 205-361 μg/cigarette in the sidestream smoke [18]. Similarly, smoke of other tobacco products also contains BD. Bidi, an indigenous form of cigarette in South Asian countries, was reported to produce 63.8 μg BD/bidi in the mainstream smoke [19]. The BD yields in the mainstream smoke of 60 commercial U.S. little cigars vary from 46 to 243 μg/cigar [20].

A source that may be important for certain microenvironments is smoke released by cooking oils at high temperature. It has been known that heating of cooking oil releases BD [1]. The BD levels released depend on the types of cooking oil and temperature [21, 22]. For example, the vapor of unrefined Chinese rapeseed oil heated to 275 °C contains ~500 μg/m^3^ of BD, which is 4- and 14-fold higher than that in the vapor generated at 240 and 185 °C, respectively. This BD concentration is also 9- and 22-fold higher than that in the vapor released from heated soybean oil and peanut oil, respectively [21]. The smoke released during cooking Sichuan, Cantonese, and Shanghai cuisine contains BD at 12.87, 4.53, and 4.20 μg/m^3^, respectively [23].

In recent years, many newly-identified sources have been reported, including emissions from oil and natural gas extraction industry [24], pyrolysis of waste plastics [25], pulp and paper industry [26], domestic waste landfill sites [27], household laser processing machines [28], and emissions from operations during electrosurgery [29–32], and from plants [33] and a soil bacterium species [34]. Among them, two types of sources are worth special attention. One of them is the surgical smoke generated during electrosurgery, which contains exceedingly high concentrations of BD (up to 42 mg/m^3^ or 19.06 ppm [29]) and thus may pose great cancer risks for operating room personnel [29–32]. The other is the natural sources; recently it was reported that BD was one of the dominant non-methane hydrocarbons generated by *Pinus massoniana* and *Schima superba*, two tree species native to southern China, with the emission rates varying from 10 to 65 nmol (0.54 to 3.5 μg)/g dry leaves/h [33]. BD was also reported to be generated by *Bacillus artrophaeus* LSSC22, a soil bacterium strain, and be able to inhibit proliferation and chemotaxis of *Ralstonia solanacearum* (*Rsc*) TBBS1, the phytopathogen causing bacterial wilt disease in tobacco [34]. These studies are the first reports on the natural sources of BD.

Because automobile exhaust contributes a majority of the BD emissions in the environment, BD is generally accepted as a mobile source pollutant or a traffic-related pollutant [35, 36]. Nonetheless, several population-based studies have revealed that environmental tobacco smoke (ETS) is a primary contributor to human BD exposure (see below). In other words, ETS is the main source for the general population in terms of human exposure. The observation is important for epidemiological studies.

### The airborne concentrations of BD

The BD concentrations in the ambient air vary widely and are dependent on locations or sites, which can be roughly divided into two categories: industrial/industry-related sites and non-industrial sites. Obviously, the latter is more important for the general population.

Unsurprisingly, the BD concentrations at industrial and industry-related sites, e.g., sites close to industrial facilities, are usually higher in comparison with those at non-industrial sites [35]. For example, a maximal BD concentration of 27 ppb (60 μg/m^3^) was observed in a county downwind of a refinery facility in the Industrial Heartland of Alberta, Canada’s largest hydrocarbon processing center [37]; an overall arithmetic mean of 120 μg/m^3^ was reported for various industries in Italia during 1996-2015 [38]. Gallego et al. reported that the BD concentrations in a few Catalan urban areas in Spain near petrochemical facilities ranged from 15 to 33 μg/m^3^ [39]. A study to measure VOCs at 16 sampling sites in the North Industrial Complex of Tarragona, Spain, reported a BD range from 0.31 to 15.19 μg/m^3^ [40].

The BD concentrations at non-industrial sites vary greatly. Huy et al. compiled the BD concentrations in different countries and regions reported in the literature [41]. The average concentrations range from 0.01 to 3.3 μg/m^3^, but the developed countries or regions (e.g., Hong Kong) have lower concentrations (0.01-0.91 μg/m^3^) compared to the developing countries (0.35-3.3 μg/m^3^) [41]. An EPA official document released in 2012 provided the range of the mean BD concentrations in U.S. cities and suburban areas, and the average background concentration, which were 0.1-2 and 0.13 μg/m^3^, respectively [42]. The BD concentrations in rural areas (0.002-0.125 μg/m^3^) are typically one order of magnitude lower than those in urban areas [35, 41]. Furthermore, in urban areas, the BD concentrations at commercial sites, particularly at sites close to the street level in the urban core, are usually higher than those at residential sites [35]. A modeling study of the BD concentrations in Minnesota, U.S., showed that the concentrations were the highest in the center of the metro area and decreased with distance from there [43]. Technological advances have led to significant decreases in the ambient air levels of BD and other VOCs in developed countries [35, 44–47].

However, in terms of human exposure, the BD concentrations in microenvironments, particularly indoor microenvironments, where people spend most of time, are more important than those in the ambient environment. Importantly, the BD concentrations in microenvironments are often unrelated to the ambient measurements at nearby monitors [48], which mostly reflect the BD concentrations in near-road environments [36]. A recent report also found that there was no association between ambient VOC levels, including BD, and personal exposures [44].

The BD concentrations in microenvironments vary widely depending on the proximity to emission sources and their magnitudes. Automobile exhaust and ETS are the major sources of BD at non-industrial sites, as a result, the microenvironments with these sources often show relatively high BD concentrations. Indeed, the roadside BD levels are usually higher than the background levels [14, 35, 36]. Exhaust from biomass burning is also an important source of BD in the environment, consequently, some special microenvironments with the source can have elevated BD levels. Temples are one of such microenvironments due to incense burning; an air BD concentration as high as 10.46 μg/m^3^ inside three temples in Thailand has been reported [49]. Important microenvironments concerning human exposure include:

1) Vehicles. In a study to investigate the concentrations of VOCs in urban domestic and public microenvironments, Kim et al. found that the mean BD concentration in automobile was 7.9 μg/m^3^, which was the highest in all microenvironments examined [50]. Even the air surrounding moving vehicles contains relatively high concentrations of BD; a mean of 3.0 μg/m^3^ and a maximum of 6.9 μg/m^3^ have been reported [1].

2) Rooms with smoking. Cigarette smoke contains significant amounts of BD, as a result, the BD concentrations in smoke-filled rooms can be high. An experiment, which was performed in a ventilated 18 m^3^ laboratory chamber with 6 cigarettes being smoked, showed that the BD concentrations were 122, 34, and 3.9 μg/m^3^ as measured at 20 min, 2 h, and 18 h after smoking had ended, respectively [51]. Before a smoking ban was implemented in Ireland, the average BD concentration in pubs was reported to be 4.15 μg/m^3^ [52]. A mean BD concentration of 1.7 μg/m^3^ in smoking homes, which is 3.4 times higher than that in nonsmoking homes, has been reported [50].

3) Certain restaurants and kitchens. Recently, Huang et al. reported that the dining area in a Chinese hot-pot restaurant had a BD concentration of 7.73 μg/m^3^, probably due to the use of gas stoves [23]. Kitchens, especially those cooking Chinese cuisine by using high-temperature oils, can be a potential microenvironment with relatively high BD concentrations due to the BD release from heated oils.

4) Operating rooms. Because the surgical smoke generated during electrosurgery contains extremely high concentrations of BD [29], plus most surgeons do not use smoke management at all [30, 32] or the mobile smoke evacuation systems cannot effectively remove BD in the smoke [29], the operating rooms performing electrosurgery can be an indoor microenvironment with relatively high BD concentrations.

### Human exposure to BD

Human exposure to BD can be divided into two categories: occupational and non-occupational. Occupational exposure usually occurs at industrial sites, and the exposure levels are generally high but are dependent on the types of industry, activity sectors, and occupational groups. An investigation on Italian working force indicated that the exposure levels in most activity sectors or occupational groups ranged between 10 and 200 μg/m^3^, but the exposure levels in the manufacture of rubber and plastic products (320-360 μg/m^3^), and the manufacture of coke and refined petroleum products (340-390 μg/m^3^) were significantly higher than those in other sectors [38]. The firm size can influence the exposure levels as well; micro- and small enterprises have greater probability to show higher exposure levels [38]. In a petrochemical plant in Iran, the BD exposure level reaches 560.82 ± 811.36 μg/m^3^ [53]. However, technical advances and changes in the operating practices can greatly reduce the human exposure. For example, two studies published in 2016 and 2017 reported low exposure levels in the Swedish petroleum refinery industry, which varied from 0.3 to 22.4 μg/m^3^, depending on the occupational groups [54, 55]. Similarly, a study published in 2017 reported that the BD exposure level in the vicinity of a major petrochemical complex in Thailand was as low as 0.04 μg/m^3^ [56].

Recently, some newly-identified occupations outside the common activity sectors are found to have low to moderate BD exposure levels, which include underground coal miners (~2.1 μg/m^3^) [57], firefighters (23.6 μg/m^3^), police forensic investigators (9.68 μg/m^3^) [58], and hairdressers [59]. It is noted that exposure of hairdressers to BD was assessed through the levels of urinary BD biomarkers rather than through measuring the airborne BD concentrations; the median concentration of a BD biomarker among hairdressers, who were all female, was found to be more than 5 times higher compared to women in the general population [59].

On the other hand, non-occupational exposure to BD is widespread and microenvironments can be the primary determinant. Because humans usually spend most of time indoors rather than outdoors, human exposure to BD is mainly dependent on the concentrations in indoor microenvironments. Kim et al. determined personal exposures of 12 urban dwellers to VOCs by direct measurements via personal monitoring and discovered that exposure at home contributed to 51-87% of overall individual exposure to BD [60]. Similarly, Huy et al. reported that exposure at home dominantly contributed to the total cancer risk caused by BD (56-86%) [41]. In another study, Du et al. estimated that ~70% of the overall cancer risks caused by 16 HAPs (including BD) in China was attributed to exposure to these pollutants at home [61]. In a recent population study, Konkle et al. found that, although the ambient air concentrations of 11 VOCs in the United States, including BD, decreased from 2005 to 2013, all corresponding urinary metabolites of the VOCs increased over approximately the same timeframe except for one metabolite [44]. As pointed out by the authors, the finding indicated that these VOCs in the ambient air were not the major source of VOC exposure [44], thus providing indirect support for the role of microenvironments in exposure to BD and other VOCs.

For non-occupational exposure to BD, ETS can be a principal contributor. Measurements of the BD concentrations in indoor air before and after the implementation of a smoking ban in Ireland provide direct evidence of the contribution of ETS to the BD levels in indoor microenvironments. The airborne BD concentration prior to the implementation of the ban, which was 4.15 μg/m^3^, dropped down by nearly 20-fold to 0.22 μg/m^3^ after the ban was fully implemented [52].

As mentioned above, BD is generally considered an automobile source pollutant or a traffic-related pollutant, however, the results from several population studies suggest that human exposure to BD is mostly attributed to ETS. In a study to investigate biological monitoring of exposures to ETS in the general population, Aquilina et al. observed that urinary concentration of cotinine, a well-known ETS biomarker, showed significant correlation with the individual exposure to airborne BD that was measured with personal exposure samplers [62]. The finding suggests that ETS is a significant source of exposure to BD for the general population. In a study assessing exposure to VOCs among pregnant women in the United States, Boyle et al. found that smoking was positively associated with the metabolite levels of BD [63]. The results obtained from a recent study on nearly 6,000 participants indicated that tobacco smoke was a major source of BD exposure in the general U.S. population [64]. These reports are consistent with the evaluation for residential exposures of U.S. non-smokers based on material-balance modeling; the evaluation indicated that ETS was the dominant source of environmental inhalation intake for BD, which was estimated to be 16-37 μg/day [65]. Taken together, for the general population, it appears that the indoor microenvironments are the dominant factor in human exposure to BD, and ETS is the primary source of BD in these microenvironments.

A special microenvironment that might lead to relatively high exposure to BD is kitchens with the cooking practice using high-temperature oils. However, the studies concerning the microenvironment have been extremely scarce. Using the levels of urinary biomarkers as the metric to assess exposure to VOCs, an investigation on Chinese women who regularly cook at home has failed to provide support for the women to experience elevated BD exposure. However, the size of the study is quite small and many confounders can have influenced the outcome [66].

Interestingly, exposure to BD was found to be dependent on socio-demographic characteristics. The populations with a high percentage of ethnic/racial minorities and low income tend to have higher exposure levels [67].

### The cancer risks of BD

BD has high cancer risks. The inhalation unit cancer risk determined by EPA is 3 × 10^-5^ per μg/m^3^ or 0.08 per ppm, in other words, 0.03 μg/m^3^ of BD causes a benchmark cancer risk of 1 × 10^-6^ [1, 68]. Because the ambient BD concentrations in most urban areas usually range from approximately 0.1 to 1 μg/m^3^, the cancer risk for the general population in cities is around 10^-5^ [41]. This has been demonstrated by many studies [24, 61, 69–73]. The concentrations of BD in small cities and rural areas are usually lower, as a result, the cancer risks are lower (approximately 10^-6^) [74, 75]. On the other hand, the cancer risks at industrial sites or in areas with industrial point sources of BD are usually high [76]; the risks at heavily-polluted sites or those caused by occupational exposure can even reach 10^-3^ [27, 53, 77]. Due to its high inhalation unit cancer risk, BD is often a major contributor to the total cancer risks caused by VOCs in industrial sites. For example, an investigation on process-specific emission characteristics of VOCs from petrochemical facilities in the Yangtze River Delta, China, found that the process unit producing BD had the largest cancer risk [78].

Because human exposure to BD is dominantly attributed to ETS, the cancer risks caused by residential exposure are usually greater than those caused by the ambient air. The cancer risks caused by residential exposure for U.S. non-smokers have been estimated to be 4.2 × 10^-5^-5.3 × 10^-4^ by using material-balance modeling [65].

BD is one of the environmental pollutants with the highest cancer risks. In almost all studies to investigate the cancer risks of pollutants, BD has been ranked as one of the top pollutants. In 2007, a U.S. study showed that among 17 pollutants, benzene, formaldehyde, and BD were the top three pollutants with the cancer risks on the order of 10^-5^-10^-4^ [79]. In another study, McCarthy et al. used the EPA national ambient air quality data for the period 2003 through 2005 and found that among 65 air toxics, concentrations of benzene, carbon tetrachloride, arsenic, BD, and acetaldehyde exceeded the 10^–6^ benchmark level at most sites in the United States [80]. In an investigation conducted in Tianjin, China, benzene, BD, and chloroform were listed as the top three pollutants among 10 monitored VOCs [81]. Du et al. compiled the data of 16 HAPs mostly from 2003 to 2013 in urban areas of China, and concluded that formaldehyde, 1,4-dichlorobenzene, benzene, and BD were the major risk contributors, which yielded the highest cancer risks (all > 10^-5^) [61]. Dhaini et al. investigated the cancer risks of air pollutants in Beirut, Lebanon, and discovered that benzene and BD were the major contributors, which accounted for 39-43% and 25-29% of the cumulative risks, respectively [71]. In Xi’an, China, formaldehyde, BD, and 1,2-dichloroethane were found to be the top three contributors to the cancer risks [70]. In two coastal cities in Metro Vancouver, Canada, the top pollutants were determined to be carbon tetrachloride, benzene, and BD [72]. In Calgary, another Canadian city, the same three pollutants were also listed as the top contributors to the cancer risks [24].

### The metabolism of BD

BD is an indirect carcinogen, i.e., it must be biotransformed into metabolites to exert its mutagenicity/carcinogenicity. Actually, non-carcinogenic effects of BD are also considered to be mediated by its metabolites [1].

**Fig. 1:**
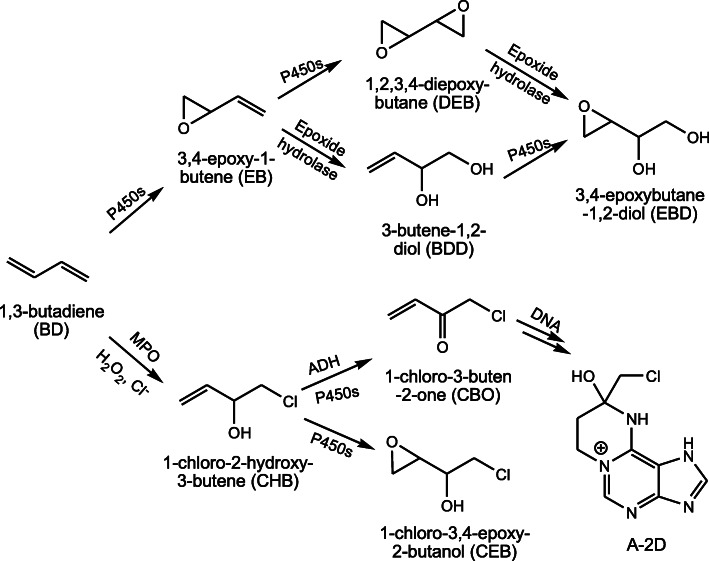
The metabolic pathways of BD. The P450-mediated metabolism is an established pathway and the MPO-mediated one is a proposed pathway. (P450s, cytochrome P450 enzymes; ADH, alcohol dehydrogenase; MPO, myeloperoxidase; GSH, glutathione)

It has long been known that BD can be metabolized to form 3,4-epoxy-1-butene (EB) by cytochrome P450 enzymes (P450s), which can be further biotransformed into 1,2,3,4-diepoxybutane (DEB) by P450s, or into 3-butene-1,2-diol (BDD) by epoxide hydrolase (Fig. 1). BDD can be converted to 3,4-epoxybutane-1,2-diol (EBD) by P450s [1, 6], which was recently reported to undergo further bioactivation to form a bifunctional epoxy aldehyde [82]. DEB can also be converted to EBD by epoxide hydrolase. The three epoxides, EB, DEB, and EBD, can readily react with nucleosides and DNA to yield DNA adducts, and are genotoxic and mutagenic [7, 83–87]. *In vivo*, BDD and EBD are the most abundant metabolites, and DEB is the metabolite with the lowest concentrations [6, 7, 88].

Because of the presence of two epoxy moieties in the molecule, DEB not only has stronger reactivity in comparison with EB and EBD [89–91], but also can form DNA cross-links [7, 83, 92–96] and DNA-protein cross-links [97]. As a result, clastogenicity of DEB is very high; among ~100 IARC carcinogens (Group 1, 2A, and 2B), DEB shows highest level of micronucleus (MN) induction in mice [98]. DEB is the most genotoxic and mutagenic metabolite among the three epoxides with the relative potencies of DEB >> EB > EBD [6, 7]. However, it should be noted that the relative potencies can be different when considering the stereochemistry of the metabolites. A specific EBD stereoisomer, (2*R*,3*S*)-EBD, has been found to be at least 30-fold more mutagenic than the other three EBD stereoisomers [99]. Importantly, the mutagenic potency of (2*R*,3*S*)-EBD is 10-to-20-fold greater than EB stereoisomers and is only 5-to-10-fold less than DEB stereoisomers [99].

An alternative metabolic pathway has been proposed, in which BD is converted to 1-chloro-2-hydroxy-3-butene (CHB) in the presence of hydrogen peroxide and chloride anion (> 50 mM) by myeloperoxidase (MPO), a peroxidase almost specifically present in neutrophils and monocytes [100–102]. CHB, a chlorohydrin, can be converted to 1-chloro-2-buten-2-one (CBO) and 1-chloro-3,4-epoxy-2-butanol (CEB) by P450s or alcohol dehydrogenase (ADH) [103–105]. CBO, a strong Michael acceptor, rapidly reacts with glutathione (GSH) to yield GSH conjugates [103], and can also readily react with nucleosides and DNA to form multiple DNA adducts [106–109], among which an adenine adduct, A-2D (Fig. 1), was detected in cells treated with low concentrations of CBO [109]. Importantly, CHB is mutagenic as determined by the Ames test, and CHB, CBO, and CEB are all genotoxic as assessed by the comet assay with CBO being much more potent than CHB, CEB, and also DEB [105, 110].

The alternative metabolic pathway may exist in the bone marrow and blood, which contains abundant neutrophils. Preliminary data showed that the MPO-mediated metabolism of BD could occur in murine neutrophils and fresh human blood [111]. Thus, it has been proposed that this pathway may play a role in carcinogenesis of BD in *humans* [101, 102], because the lymphohematopoietic system, i.e., the bone marrow, is the target organ for BD in humans on the basis of the epidemiological studies [1, 68].

Although it has been established that the mutagenic and carcinogenic effects of BD are caused by its genotoxic metabolites, the specific metabolite(s) responsible for the effects are still in debate. Among the three epoxides, DEB is often considered to be the ultimate culprit responsible for BD mutagenicity/carcinogenicity [95, 112, 113] due to its highly genotoxic and mutagenic potency compared to EB and EBD [6, 7, 83, 84]. On the other hand, EB and EBD have also been proposed to be the predominant cancer-initiating metabolites due to their much higher *in vivo* concentrations (especially EBD) compared to DEB [114], and the role of EBD has been implicated through experiments in BDD-dosed mice and rats [87].

Making the issue more complex, what metabolite(s) are responsible for BD mutagenicity/carcinogenicity appears to be species- and BD concentration-dependent [87]. Specifically, the culprits are considered to be DEB in mice, and EB and/or EBD in rats; or, DEB and EBD are considered responsible for the toxic effects of BD at low and high BD levels, respectively [87, 114]. For humans, the issue remains to be elucidated but DEB is thought to contribute little to BD carcinogenicity and EBD may cause the greatest risk [115, 116]. Another study estimates that DEB and EBD contribute 7% and 92% to the total genotoxic dose in humans, respectively [117]. Consistent with the observation is the finding that treosulfan, a chemotherapeutic agent that is non-enzymatically converted to DEB as its bioactive form [118], induces different histological subtypes of lymphohematopoietic neoplasms (acute myeloid leukemia) from those caused by occupational exposure to BD. Therefore, it has been speculated that monoepoxide metabolites may play a more important role than DEB in BD carcinogenesis in humans [119].

### Urinary biomarkers of BD

#### Urinary BD biomarkers reported in the literature

EB, DEB, EBD, and CBO (Fig. 1) are reactive metabolites and can react with proteins (e.g., hemoglobin) and DNA. The products formed, i.e., hemoglobin and DNA adducts, can be used as biomarkers [120, 121]. Meanwhile, these metabolites can also undergo biotransformation via the mercapturic acid pathway to yield *N*-acetyl-L-cysteine (NAC) conjugates, which are excreted in urine and can be used as biomarkers as well. BDD and CHB need to be converted to other intermediates, presumably hydroxymethyl vinyl ketone and CBO, respectively, before biotransformation via the mercapturic acid pathway.

However, to detect hemoglobin and DNA adducts, blood and tissue samples are usually needed, which greatly restricts the application of these biomarkers in studies involving human subjects due to the difficulty in collecting the specimens. By contrast, urine is readily available and has fewer biohazard concerns. Moreover, a large proportion of absorbed BD (usually 30-60% as observed in mice, rats, and monkeys) is excreted in urine as biotransformed products [122, 123], therefore, the amounts of the biotransformed products in urine are expected to be larger than those in other biological matrices (e.g., blood and tissues). As a result, urinary BD biomarkers have been widely used, particularly in studies concerning human subjects. Thus, in this section, we will only review progress in urinary BD biomarkers.

**Fig. 2:**
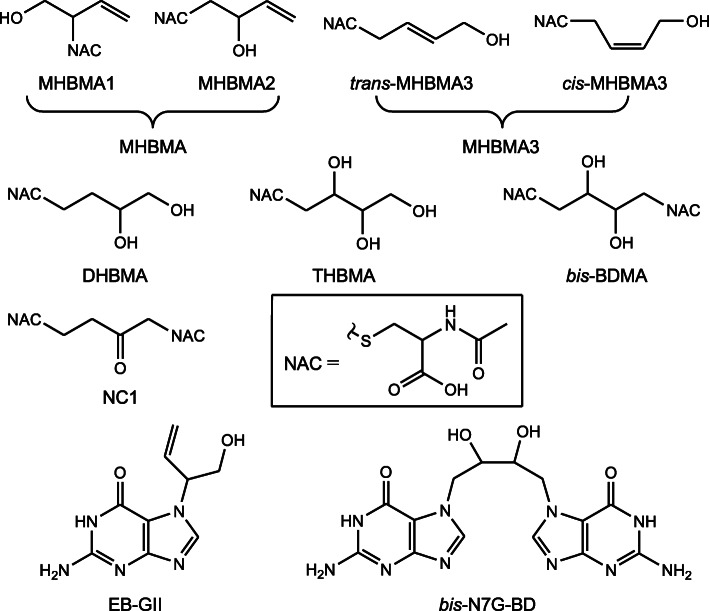
The structures of the urinary BD biomarkers reported in the literature. MHBMA1, *N*-acetyl-*S*-[1-​(hydroxymethyl)​-​2-​propen-​1-​yl]​-L-cysteine; MHBMA2, *N*-acetyl-*S*-(2-hydroxy-3-buten-1-yl)-L-cysteine; *trans*- and *cis*-MHBMA3, *trans*- and *cis*-*N*-acetyl-*S*-(4-hydroxy-2-buten-1-yl)-L-cysteine; DHBMA, *N*-acetyl-*S*-(3,4-dihydroxybutyl)-L-cysteine; THBMA, *N*-acetyl-*S*-(2,3,4-trihydroxybutyl)-L-cysteine; *bis*-BDMA; 1,4-*bis*(*N*-acetyl-L-cystein-*S*-yl)butane-2,3-diol; NC1, 1,4-*bis*(*N*-acetyl-L-cystein-*S*-yl)-2-butanone; NAC, *N*-acetyl-L-cysteine; EB-GII, *N*7-[1-​(hydroxymethyl)​-​2-​propen-​1-​yl]guanine; *bis*-N7G-BD, 1,4-*bis*(gua-7-yl)-2,3-butanediol. MHBMA1 and MHBMA2 are collectively called monohydroxybutenyl mercapturic acid (MHBMA), and *trans*- and *cis*-MHBMA3 are similarly called MHBMA3.

Through the mercapturic acid pathway, EB is primarily biotransformed into two regioisomers, *N*-acetyl-*S*-[1-​(hydroxymethyl)​-​2-​propen-​1-​yl]​-L-cysteine (MHBMA1) and *N*-acetyl-*S*-(2-hydroxy-3-buten-1-yl)-L-cysteine (MHBMA2) (Fig. 2) (it should be noted that each of the two regioisomers consists of two diastereomers), which are collectively called monohydroxybutenyl mercapturic acid (MHBMA) [2, 124, 125]. In addition, a small fraction of EB can undergo a rearrangement of double bond to yield *N*-acetyl-*S*-(4-hydroxy-2-buten-1-yl)-L-cysteine (configurations unspecified, presumably *trans*-), a trace product usually called MHBMA3 [126, 127]. The corresponding biotransformation products of BDD, EBD, DEB, and CHB are *N*-acetyl-*S*-(3,4-dihydroxybutyl)-L-cysteine (DHBMA), *N*-acetyl-*S*-(2,3,4-trihydroxybutyl)-L-cysteine (THBMA), 1,4-*bis*(*N*-acetyl-L-cystein-*S*-yl)butane-2,3-diol (*bis*-BDMA), and 1,4-*bis*(*N*-acetyl-L-cystein-*S*-yl)-2-butanone (NC1) (Fig. 2), respectively [103, 104, 113, 125, 128, 129].

DNA adducts can be released from DNA spontaneously or via the DNA repair process. The DNA adducts released are eventually excreted in urine and thus can also be used as urinary biomarkers. Although many BD-derived DNA adducts have been identified [7], so far only two DNA adducts, *N*7-[1-​(hydroxymethyl)​-​2-​propen-​1-​yl]guanine (EB-GII) and 1,4-*bis*(gua-7-yl)-2,3-butanediol (*bis*-N7G-BD), have been used as urinary biomarkers [130, 131].

#### MHBMA and DHBMA

MHBMA and DHBMA are the classic biomarkers of BD and have been used for almost 30 years [124, 132, 133]. They are the major metabolites of BD in urine with the sum of the amounts constituting 50-90% of the total amounts of all urinary metabolites in different species (mouse, rat, hamster, and monkey) [125]. In humans, DHBMA is the most abundant metabolite in urine and is readily detected [113, 132]. MHBMA is more sensitive that DHBMA [133–136]. The two biomarkers, especially DHBMA, have been used in population-based studies [44, 64, 136].

Due to the difficulty in chromatographic separation of the two regioisomers, in most studies the sum of the amounts of MHBMA1 and MHBMA2 is used as a single biomarker (i.e., MHBMA) [124, 135–138]. In some studies, the two regioisomers are separated and the concentrations of individual regioisomers are reported. The data obtained show that MHBMA2 is the dominant isomer in human urine [64, 139].

#### MHBMA3

MHBMA3 is a biomarker that is worth special attention. The metabolite was first discovered in 1995 through the reaction of EB with NAC under reflux; its structure was characterized but the configuration (*trans*- or *cis*-) was not determined. However, this product failed to be detected in urine of rats and mice administered EB [126]. It turned out to be an issue of sensitivity of the instrumental method, because in a subsequent study, Richardson et al. successfully detected the metabolite in urine of rats and mice administered ^14^C-labeled EB. Nonetheless, MHBMA3 was a trace metabolite with the amounts being only 0.5% of the administered doses or even less [127]. In 1999, the metabolite was detected in urine of mice exposed to ^14^C-labeled BD but accounted for only 0.1% of the administered doses, however, it was virtually not present in urine of rats under the same BD exposure [128]. It is noted that in the experiment, the amount of MHBMA was at least 120-fold greater than that of MHBMA3 [128].

Probably due to its tiny amounts in urine of rats and mice, no studies concerning MHBMA3 have been published over the following decade. The next study involving the metabolite was published in 2012, when Alwis et al. detected MHBMA3 in human urine for the first time [139]. It is worth noting that the urine samples were collected from the general U.S. population, rather than the subpopulation with high exposure (workers occupationally exposed to BD). Furthermore, it is unexpected that MHBMA3 turned out to be the most abundant metabolite in human urine among the three EB-derived metabolites (MHBMA1, MHBMA2, and MHBMA3, which were quantitated separately) with the concentrations being 36 ± 34 and 6.40 ± 10 ng/mL for smokers and non-smokers, respectively. Surprisingly, MHBMA3 had a very high frequency of detection (99%). By sharp contrast, MHBMA1 failed to be detected in a vast majority of samples (either smokers or non-smokers) and MHBMA2 was detected only in urine of smokers but its concentrations were very low [the mean concentration was 1.80 ± 2.10 ng/mL with the limit of detection (LOD) being 0.70 ng/mL] [139]. The finding was confirmed by a recently published study with 5,897 participants, in which the frequency of detection of MHBMA1 and MHBMA2 in urine samples was only 0.7% and 9.8%, respectively [64]. This is opposite to the observation in animal experiments, in which MHBMA1 and MHBMA2 were overwhelmingly dominant metabolites formed, and MHBMA3 could hardly be detected [128].

Following the report of Alwis et al., more than 10 studies on the general population using MHBMA3 as the BD biomarker have been published, with most studies being conducted in the U.S. populations [44, 63, 64, 140–153]. These studies performed in the general U.S. populations showed similar results to those reported by Alwis et al. [139]. However, the results obtained in the populations in other countries and regions are different. Chiang et al. reported that the mean MHBMA3 concentrations in 55 Taiwanese smokers and 55 non-smokers were 129.2 ± 99.2 and 52.3 ± 36.8 ng/mL, respectively [140], which were considerably higher than that reported by Alwis et al., in particular, the concentrations in non-smokers. A recently published study on the general population in Wuhan, China, reported a surprisingly low frequency of detection at only 9.4% [152]. Similarly, another study performed in China even failed to detect MHBMA3 in 100 urine samples from children aged 6-12 years [153]. A possible explanation for the surprising observations reported in the two studies is that the authors used *cis*-MHBMA3 as the reference standard and the LOD in the latter study was somewhat high (3.13 ng/mL) [152, 153], although in the studies of Alwis et al. [139] and other authors [44, 63, 64, 140–148, 151], the configuration of the MHBMA3 standard was not specified.

MHBMA3 is a sensitive BD biomarker. It can clearly distinguish non-smokers from smokers. In fact, all population-based studies consistently show that there are statistically significant differences in the MHBMA3 concentrations between smokers and non-smokers [63, 64, 139–142, 149, 151]. Quitting smoking can be reflected in the change in the MHBMA3 level; in a study on over 1,100 adult exclusive daily cigarette smokers, a dramatic reduction in the MHBMA3 level was observed among those quitted tobacco use entirely [147]. The biomarker can also indicate exposure to ETS; in an investigation on exposure of non-smokers to VOCs from secondhand smoke, St. Helen et al. observed that the MHBMA3 concentrations exhibited the greatest increase (2.1-fold) from 0 to 8 h postexposure among 9 VOC biomarkers [154]. In addition, MHBMA3 can even discriminate between duel cigarette/e-cigarette users and exclusive cigarette smokers [141]. Furthermore, the biomarker can distinguish among light, average, and heavy smokers. In Wave 1 of the Population Assessment of Tobacco and Health Study, the biomarker data of more than 2,700 U.S. adult daily cigarette smokers were analyzed and it was observed that the concentrations of biomarkers, including MHBMA3, consistently increased with cigarettes smoked per day (CPD) [141]. In the 2011-2016 U.S. National Health and Nutrition Examination Survey, by examining the urinary BD biomarkers, including MHBMA1, MHBMA2, MHBMA3, and DHBMA, in 5,897 participants, Nieto et al. observed clear dose-response relationships between the MHBMA3 or DHBMA concentrations and CPD [64]. Specifically, compared to non-smokers, smoking 1-10, 11-20, and > 20 CPD was significantly associated with 475%, 849%, and 1,143% higher MHBMA3 levels, respectively (all *p* < 0.0001). However, the corresponding increases for DHBMA were only 32.8%, 44.2%, and 102%, respectively [64]. In addition, another study showed that the increase in number of days that the tobacco products were used during the last five days was associated with increased levels of MHBMA3 (*p* < 0.01) but not DHBMA [149].

Besides smokers and non-smokers, significant differences in the MHBMA3 levels have also been observed among different subpopulations, including those with different gender, ethnic/racial group, and age, although the results are not always consistent. For example, it has been discovered that the MHBMA3 levels in female smokers were significantly high than those in male smokers [64, 142, 149]. When comparing differences among different ethnic/racial groups, the MHBMA3 levels in non-Hispanic white smokers were observed to be higher than those in non-Hispanic black ones [149]. Another study also found that the MHBMA3 levels in non-Hispanic white were significantly higher than those in non-Hispanic black and Hispanic [142]. However, the differences failed to be observed in a recently published study [64].

The differences among subpopulations with different ages were consistently observed by three groups of authors. Jain reported that there was a statistically significant positive association between the MHBMA3 level and age [149], and specifically, children aged 6-11 years old had significantly higher MHBMA3 level compared to non-smoker adults aged ≥ 20 years [150]. De Jesus et al. found that after controlling for tobacco use and other cofactors and using participants’ age of 25–34 years as the reference, young adults (18-24 years) had significantly lower MHBMA3 levels, but older adults (≥ 55 years) had significantly higher levels (all *p* ≤ 0.0001) [142]. Similar results were also obtained in a recent study reported by Nieto et al., although in this study, age was categorized into somewhat different ranges from the preceding study. In this study, Nieto et al. reported that the MHBMA3 concentrations in older adults (aged 40-59 and ≥ 60 years) were significantly higher compared to those in younger adults (aged 20-39 years) [64]. Among all participants (*n* = 5,897) and the participants who did not use tobacco products (*n* = 5,171), with the exception of adolescents (aged 12-19 years), all age groups, including children (aged 3-5 and 6-11 years), had higher MHBMA3 levels compared with young adults (aged 20–39 years) [64], which is consistent with the observation reported by Jain [150].

A major issue concerning MHBMA3 is that its configuration is not specified in all studies except for two recent ones [152, 153]. This compound has two configurations or two stereoisomers, i.e., *trans*- and *cis*-MHBMA3 (Fig. 2), which should be well separated chromatographically. Unlike MHBMA, which is the mixture of MHBMA1 and MHBMA2 and is usually used as a single biomarker in most studies, the researchers in MHBMA3-related studies did not describe that this compound was a mixture of two stereoisomers. Moreover, the MHBMA3 standards available commercially are either *trans*- or *cis*-isomer. Therefore, MHBMA3 detected in human urine should be one of the two stereoisomers rather than the mixture of the two stereoisomers. It has not been clear whether the difference in the frequency of detection of MHBMA3 between the studies of Qian et al. or Kuang et al. [152, 153] and those reported by other authors [44, 63, 64, 139–148, 151] is caused by the configuration of the biomarker.

#### THBMA

THBMA was tentatively identified first in urine of mice exposed to BD [155] and was confirmed later by using animals exposed to ^14^C-labeled BD [128]. Interestingly, the metabolite was not detected in urine of rats and mice administered ^14^C-labled EB [127]. In 2000, van Sittert et al. tried to detect THBMA in human urine but the attempt was unsuccessful due to interferences from the sample matrix [124]. Until 2011, the Tretyakova laboratory was able to develop an LC-MS/MS method to detect the metabolite in human urine, whose concentrations in smokers and non-smokers were reported to be 21.6 and 13.7 ng/mg creatinine with the difference being statistically significant (*p* < 0.01), respectively. Furthermore, the THBMA concentrations in urine declined 25-50% following smoking cessation [129]. The biomarker was also detected in urine of workers occupationally exposed to BD with the mean concentration being 157 ng/mg creatinine [113]. A subsequent study on workers in Czech Republic observed statistically significant differences not only between exposed workers and the control, but also between the male control and the female control. The MHBMA concentrations in the male control were significantly higher than those in the female control (57.1 ± 33.5 vs. 24.2 ± 16.6 ng/mL, respectively) even though both control groups were exposed to the same ambient BD levels (7 ± 5 μg/m^3^) [135].

#### bis-BDMA

*Bis*-BDMA was first synthesized in 2014 by the Tretyakova group and was used as a biomarker [113]. In rat urine following exposure to 200 ppm (442 mg/m^3^) BD for two weeks, the mean *bis*-BDMA concentration was 4.8 ± 2.9 μg/mL, which was 16-, 44-, and 31-fold lower than those of MHBMA, DHBMA, and THBMA, respectively. However, this biomarker was not detected in urine of smokers and workers occupationally exposed to BD [113]. In urine of mice exposed to 590 ppm (1,300 mg/m^3^) BD for two weeks, the *bis*-BDMA concentration was 8.09 ± 6.3 μg/mL [131].

#### NC1

NC1 is the urinary biomarker of CHB, the potential BD metabolite formed via the alternative MPO pathway. It was first synthesized in 2017 [111, 156] and subsequently was detected in urine of CHB-administered rats and mice [104]. Whether the biomarker can be detected in urine of animals and humans exposed to BD has not been reported.

#### EB-GII

EB reacts with the guanine residues in DNA at the *N*7-position to form two products, *N*7-(2-​hydroxy-3-buten-​1-​yl) guanine (EB-GI) and EB-GII [157]. The two adducts have been detected in EB-treated cells in culture, and in tissues of rats and mice exposed to BD [157], and EB-GII has been found in human blood leukocyte DNA of smokers but below the LOD [158]. In 2017, the Tretyakova laboratory developed a highly sensitive LC-MS/MS method and was able to detect EB-GII in urine of smokers and occupationally-exposed workers [130]. The biomarker was successfully used in studies concerning humans and animals [159–161].

#### bis-N7G-BD

*Bis*-N7G-BD was first synthesized through the reaction of DEB with guanosine [92]. The adduct can be detected in tissues of rats and mice exposed to BD [95]. Use of *bis*-N7G-BD as a urinary biomarker was reported just before the review is published [131]. In the study, very low concentrations of *bis*-N7G-BD (~570 pg/mg creatinine) were detected in urine of mice exposed to 590 ppm (1,300 mg/m^3^) BD for two weeks [131].

#### Suitability of the urinary BD biomarkers for studies on the general population

Urinary biomarkers are particularly useful in large epidemiological studies because urine is much more easily available and has lower biohazard risks compared to blood. However, it should be noted that urinary biomarkers reflect recent exposures and are susceptible to variations [134]. Because several urinary BD biomarkers have been developed, a comparison of their performance can help researchers make a wise selection, especially for studies on the general population.

The urinary BD biomarkers reported can be divided into two categories: exposure biomarkers and exposure/effect biomarkers. The former includes all NAC conjugates and is surrogate biomarkers, because they only reflect the doses of internal exposure to BD or a specific metabolite (e.g., DEB). On the other hand, the latter, which includes EB-GII and *bis*-N7G-BD, is mechanistically relevant and cancer-related biomarkers, because the formation of DNA adducts is considered to be the initial molecular event in carcinogenesis.

First and foremost, selection of biomarkers is certainly dependent on the purpose of research. However, in practical applications, the selection primarily depends what subjects are used in research. Urine of laboratory animals exposed to BD usually contains high concentrations of metabolites, thus all BD biomarkers can virtually be used. Conversely, urine of human subjects in the general population is expected to contain very low concentrations of biomarkers, as a result, selection of biomarkers is quite restricted. Because urinary BD biomarkers are most useful in biomonitoring of human subjects and for laboratory animals, hemoglobin and DNA adducts can be freely selected as biomarkers, we will thus focus on the issue what biomarkers are suitable for studies on the general population.

Among the urinary BD biomarkers, MHBMA and *bis*-BDMA can easily be excluded due to either very low frequency of detection [64] or inability to be detected even in urine of occupationally-exposed workers [113]. Among other biomarkers, DHBMA, THBMA, and EB-GII have natural background that may be caused by endogenous sources, thus restricting their sensitivity [113, 124, 130, 133]. Specifically, DHBMA fails to reflect the changes in the external exposure doses in some studies [137, 162]; in particular, a study found that the DHBMA levels did not change after cessation of smoking, whereas the levels of MHBMA and the biomarkers of other VOCs rapidly decreased [137]. The difference in the THBMA concentrations between smokers and non-smokers is small (the data of smokers is only 58% higher than that of non-smokers), although it is statistically significant (*p* < 0.01) [129]. EB-GII is even worse; its concentrations in urine of smokers had no statistically significant difference from those in non-smokers [130]. However, the levels of these biomarkers exhibited significant differences between occupationally exposed workers and the controls, suggesting that they may be suitable for human subjects with high BD exposure.

Among these biomarkers, MHBMA3 stands out from the rest. Many studies already demonstrate its excellent suitability for biomonitoring exposure of the general populations to ambient BD [44, 63, 64, 140–153]. It is highly responsive to the exposure dose [64] and is well capable of reflecting exposure to ETS [154]. Compared with DHBMA, MHBMA3 exhibits stronger correlation with the serum level of cotinine, the well-known biomarker for smoking [139]. In addition, the MHBMA3 levels also show significant differences among a variety of subpopulations [64, 142, 149]. Clearly, MHBMA3 is the best urinary biomarker for biomonitoring BD exposure in the general population.

### Genotoxicity of BD in humans

Genotoxicity of BD in humans, as assessed in many molecular epidemiology studies, has been thoroughly reviewed in 2010 by Albertini et al. [7]. After that, only five studies, which were performed by two groups of Chinese researchers, have been published. Therefore, we will only provide a brief update on the issue.

The Xia group in the School of Public Health of Fudan University used the cytokinesis-block micronucleus (CBMN) assay to examine chromosomal damage in peripheral blood lymphocytes (PBL) of 166 workers in a polybutadiene latex chemical industrial plant in Ningbo, China [163]. These workers were exposed to extremely high levels of BD with the range varying from 0.05 to 1,985.99 mg/m^3^ (the median was 4.48 mg/m^3^). The mean MN frequency of the workers was significantly higher than that of the unexposed control (0.339% vs. 0.148%, *p* < 0.01), and the polymorphisms of several genes involved in bioactivation and detoxification of BD were observed to influence the MN frequency [163]. In another study, the Xia group used sister-chromatid exchange (SCE) and CBMN assays to investigate chromosomal damage in PBL of 44 BD-exposed workers in a rubber factory of Shandong, China [164]. These workers were exposed to much lower levels of BD (0.06-12.41 mg/m^3^ with the median being 1.48 mg/m^3^) compared with those in Ningbo, China. It was found that the mean MN frequency in the workers was significantly higher than that in the control without occupational exposure to BD (0.439% vs. 0.296%, *p* < 0.01), but the SCE frequency did not exhibit statistically significant difference between the exposed workers and the control. In addition, it was also observed that the polymorphisms of three glutathione *S*-transferase genes affected the MN frequency [164].

The other group of Chinese researchers, the Cao and Ao group in the Department of Hygiene Toxicology of the Third Military Medical University, Chongqing, China, conducted a 1:1 matched pair study in a population of workers in a large petrochemical facility in Nanjing, China [165–167]. The workers were exposed to significantly higher levels of BD than did the control (5.02 vs. 1.86 mg/m^3^, *p* < 0.01), and exhibited elevated frequency of MN and nucleoplasmic bridge (NPB) (both *p* < 0.01) but lower nuclear division index (*p* < 0.01) in comparison to the control [165]. The polymorphisms of some metabolism- and DNA repair-related genes, and a folate metabolism-related gene were observed to affect the NPB and nuclear bud frequency [165–167].

Additionally, it is noted that a study performed in Italy observed a statistically significant increase in the MN frequency of exfoliated buccal cells in petroleum refinery workers and also residents living in areas close to the petroleum refinery industry [168]. However, the cohort was small (*n* = 50) and the BD concentrations were not measured.

In summary, these studies indicate that BD at levels of occupational exposure is genotoxic to humans and can cause chromosome damage as assessed by the CBMN assay. However, a vast majority of studies published before 2010 have failed to observe genotoxicity of BD in humans [7]. It seems to be an issue of selection of assays and endpoints, because the previous studies examined the mutations of the hypoxanthine-guanine phosphoribosyltransferase (*HPRT*) gene, and induction of chromosome aberrations and SCE [7]. Thus, the CBMN assay appears to be the technique of choice to detect genotoxicity of BD in humans. Chromosome damage detected with the assay may be used as an effect biomarker to assess cancer risk induced by BD [169].

### Associations between BD exposure and diseases

Exposure to BD has been associated with a variety of diseases. First and foremost, as a human carcinogen, occupational exposure to BD has been causally associated with lymphohematopoietic cancers, especially leukemia [1, 2, 4]. With regard to specific forms of leukemia, the epidemiological studies of a cohort of synthetic rubber industry workers at six North American plants support an association between BD exposure and lymphoid leukemia, but not myeloid leukemia, non-Hodgkin’s lymphoma, and multiple myeloma [170–173].

BD exposure has also been associated with diseases other than lymphohematopoietic cancers, including cardiovascular disease (CVD), reproductive effects, childhood leukemia, etc. It is worth noting that children may be one of the subpopulations with greater susceptibility to the toxic effects of BD than the general population; in fact, most epidemiological studies over the last decade have been conducted on children. Thus, the studies of the adverse effects of BD on children’s health will be discussed in a separate section.

#### CVD

The association between BD exposure and CVD was first noticed in 1990. Interestingly, the association was observed only in a specific subpopulation, i.e., black male. In a study to investigate the mortality among workers at a BD facility, Divine reported a significantly elevated standardized mortality ratio (SMR) in non-white males for all causes of death, including arteriosclerotic heart disease [174]. The data from another study to investigate 12,110 male workers in eight styrene-BD polymer manufacturing plants indicated a significant excess SMR for arteriosclerotic heart disease (SMR = 1.48) in black workers than in the general population [175]. In 1996, Divine and Hartman reported updated data and similarly found that, in the cohort of 2,795 male workers at a BD facility, the non-white workers showed a statistically significant elevated SMR for all causes of death, including arteriosclerotic heart disease (SMR = 1.42) [176]. In a cross-sectional study, Shin et al. observed that BD-related VOCs decreased diastolic blood pressure but increased heart rate and brachial artery diameter, suggesting that BD and other VOCs may have rapid impacts on the human cardiovascular system [177]. In this study, more than half of 63 participants were black people (*n* = 35, 55.6%) [177].

The observation that BD exposure was associated with CVD in black male was echoed by a newly published study, in which the urinary levels of DHBMA were observed to exhibit strong associations with the urinary levels of norepinephrine and normetanephrine (two CVD risk markers) in black participants of a cohort of 346 non-smokers [148]. The result suggested that exposure to BD was associated with endothelial dysfunction and may contribute to elevated risk of hypertension in people with increased sympathetic tone, particularly in black individuals [148].

Lin et al. recently reported a positive correlation between the urinary DHBMA levels and the CVD risk factors, including carotid intima-media thickness, endothelial microparticles, and platelet microparticles [178]. However, the study was conducted in young Taiwanese.

The association between BD exposure and CVD is supported by animal experiments [179, 180]. Crotonaldehyde, a minor BD metabolite, might play a role in the etiology [181].

#### Reproductive effects

BD has been considered to have reproductive and developmental effects [182]. However, so far the effects have been observed dominantly in rodents; these effects include reduced fetal weight, fetal death, ovarian atrophy, and testicular atrophy, and the most sensitive endpoints are ovarian atrophy in female mice and testicular atrophy in male mice [1, 183]. DEB may be the critical metabolite to induce the reproductive effects [1, 182]; Dong et al. reported that DEB caused the proliferation inhibition and marked cell cycle arrest at the G2 phase but not apoptosis in mouse spermatocyte-derived GC-2 cells [86].

The studies of the reproductive effects of BD on humans have been extremely scarce. The data from an earlier study on Czech female workers exposed to BD showed no difference in pregnancy outcomes (e.g., miscarriage, still birth, ectopic pregnancies) between exposed subjects and controls. However, the numbers of the subjects and controls in this study are small (*n* = 23 and 26, respectively) [184]. In a recently published study conducted in Portland, Oregon, U.S., Willis and Hystad used vital statistics records from 2000 to 2014 (*n* = 279,051 births) to assess prenatal exposure to 19 air pollutants. They observed associations in fully adjusted models comparing the highest to lowest quintiles of exposure for certain pollutants, including the associations between BD and term birth weight [−16.86 g; 95% confidence interval (CI) = −29.66-−4.06; *p* < 0.05], and between BD and small for gestational age [odds ratio (OR) = 1.18; 95% CI = 1.07-1.30; *p* < 0.05] [185]. Another recent study indicated that there might be a potential association between BD exposure and male infertility. Poli et al. reported that the urinary DHBMA levels were negatively correlated with sperm count and sperm abnormal forms, and oxidative stress on the male reproductive tract may play an important role [186].

#### Adverse effects on pulmonary functions

Occupational exposure to high concentrations of BD may have adverse effects on pulmonary functions, including vital capacity, forced vital capacity, forced expiratory volume in the first second, and peak expiratory flow. An investigation on workers in a petrochemical plant in Iran found that compared to the control, the workers had considerably higher prevalence rates of cough, phlegm, wheezing, shortness of breath, chest tightness, and episodes of chest illness associated with cold. Furthermore, their pulmonary functions were also lower in comparison with the control and the differences were statistically significant (*p* < 0.05) [187]. However, it should be noted that the workers were exposed to high BD concentrations (the average concentration reached 560.82 μg/m^3^), although the BD levels are still below the threshold recommended by the American Conference of Governmental Industrial Hygienists [187].

### Associations between BD exposure and diseases in a specific subpopulation - children

Most epidemiological studies over the last decade to investigate the associations between BD exposure and diseases were conducted in children. Exposure to BD has been reported to show associations with several childhood diseases, including childhood leukemia, brain tumors, autism, asthma, etc. The studies are discussed below.

#### Childhood leukemia

Childhood leukemia accounts for approximately 30% of all childhood cancers in the United States, and a significant increase in the incidence has been observed in Europe and in other developed countries over the past 30 years [119]. In the meantime, the etiology of childhood leukemia remains poorly understood and few established risk factors have been identified. Over the last 20 years, there has been increasing focus on environmental pollutants, especially, the traffic-related pollutants [188–192]. As a traffic-related air pollutant and a known human carcinogen that has causally been associated with leukemia, BD has naturally received much attention in the investigations for the etiology of childhood leukemia.

The association between childhood cancers, including childhood leukemia, and exposure to a variety of air pollutants (CO, NO_x_, VOCs, dioxins, etc.) was first reported by Knox in two successional studies, in which elevated risks of childhood cancers were observed among children whose residence was near a “hot spot” of benzene or BD emissions [193, 194]. Although some increased risks could be attributable to mutual confounding, BD was found to be a powerful independent predictor [194]. For the first time, BD was identified as a specific hazard for childhood cancers, although in these studies, childhood leukemia was not singled out [193].

The finding was reinforced by subsequent studies. In an ecologic study in Texas, U.S., Whitworth et al. investigated 977 cases of childhood lymphohematopoietic cancer diagnosed from 1995-2004. The researchers examined whether the census tracts with the highest estimated levels of benzene and BD had higher incidence rates of childhood lymphohematopoietic cancer compared with the census tracts with the lowest estimated levels. Indeed, among the census tracts with the highest BD levels, they observed significantly higher rates of all leukemia [rate ratio (RR) = 1.40; 95% CI = 1.07-1.81], and elevated rates of the two most common types of childhood leukemia, acute myeloid leukemia (AML) (RR = 1.68; 95% CI = 0.84-3.35) and acute lymphocytic leukemia (ALL) (RR = 1.32; 95% CI = 0.98-1.77), but the data for AML and ALL were not statistically significant [195]. The same laboratory further investigated whether *in utero* and early life exposure to BD and other pollutants was associated with childhood leukemia, and indeed observed positive associations between exposure to BD and childhood leukemia (under the age of 5) in either single or co-pollutant models [196]. In another investigation conducted in California, U.S., Heck et al. found that in infancy, AML was positively associated with exposure to BD (OR = 2.35; 95% CI = 1.02-5.39), *o*-xylene (OR = 1.88; 95% CI = 1.02-3.45), and toluene (OR = 2.02; 95% CI = 1.03-3.94) [197].

#### Childhood brain tumors

The association between exposure to BD and childhood brain tumors has also been reported. Danysh et al. conducted a population-based study in Texas, U.S., and observed that the census tracts with medium and medium-high BD concentrations had higher astrocytoma incidence rates (RR = 1.46; 95% CI = 1.05-2.01 and RR = 1.69; 95% CI = 1.22-2.33, respectively) compared with low concentrations. Increased concentrations of BD and benzene were observed to be strongly associated with increased primitive neuroectodermal tumor (PNET) incidence rates, but the associations were not statistically significant [198]. In a subsequent study, the group evaluated the influence of residential mobility on the exposure assignment and concluded that residential mobility of children did not significantly impact the exposure assignment of BD [199]. In another study in California, U.S., von Ehrenstein et al. found that central nervous system PNETs were positively associated with interquartile range (IQR) increases in prenatal exposure to acetaldehyde (OR = 2.30; 95% CI = 1.44-3.67), BD (OR = 2.23; 95% CI = 1.28-3.88), benzene, and toluene; and also with IQR increases in exposure during the first year of life to *o*-dichlorobenzene (OR = 3.27; 95% CI = 1.17-9.14), BD (OR = 3.15; 95% CI = 1.57-6.32), and benzene. The authors concluded that *in utero* and infancy exposures to air toxics generated by industrial and road traffic sources may increase the risk of PNETs and medulloblastoma, with limited support for increased risks for astrocytoma in children up to age 6 [200].

#### Other childhood tumors

Besides childhood leukemia and brain tumors, BD exposure has also been found to be potentially associated with other childhood cancers. Heck et al. examined ambient exposure to 27 air toxics in the perinatal period in relation to retinoblastoma development with the disease diagnosed during 1990-2007 in California, U.S. They observed that retinoblastoma risk increased with pregnancy exposure to benzene (OR = 1.67; 95% CI = 1.06-2.64) and other six toxics that primarily arise from gasoline and diesel combustion and are highly correlated, including BD (OR = 1.59; 95% CI = 1.08-2.35) [201]. Prenatal exposure to air toxics, including BD, was also observed to be positively associated with malignant germ cell tumors (GCTs) in young children [202]. In a case-control study, Hall et al. identified 243 GCT cases, which were matched by birth year to cancer-free population controls (*n* = 147,100) from 1984 to 2013 in California, U.S. They observed that prenatal exposure to traffic-related air toxics during the second trimester increased GCT risk, particularly BD (OR = 1.51; 95% CI = 1.01-2.26) and *m*-/*p*-xylene (OR = 1.56; 95% CI = 1.10-2.21). A further analysis by subtype indicated elevated ORs for yolk sac tumors but no teratomas [202].

#### Childhood autism

The etiology of autism is heterogeneous and little is known about its nongenetic causes, but environmental factors have been suggested as major contributors. It has been reported that *in utero* BD exposure is associated with childhood autism [203]. In a population study, von Ehrenstein et al. identified 768 cases of autism from 148,722 children in Los Angeles County, California, U.S., during 1998-2009. They found that autism risks increased per interquartile range increase in average concentrations during pregnancy of several correlated toxics mostly loading on 1 factor, including BD (OR = 1.59; 95% CI = 1.18-2.15), *m*-/*p*-xylene (OR = 1.51; 95% CI = 1.26-1.82), and other pollutants, adjusting for maternal age, ethnicity/race nativity, education, insurance type, parity, child sex, and birth year [203].

#### Childhood asthma

In a recently published article, Kuang et al. compared the differences in several biomarker levels between asthmatic and healthy children. DHBMA was used as the biomarker of BD. The results indicated that the urinary levels of DHBMA and biomarkers of other pollutants were significantly associated with asthma (for DHBMA, OR = 2.76; 95% CI = 1.73-4.43). Oxidative stress may play an important role, because these biomarkers exhibited strong correlations with 8-hydroxy-2′-deoxyguanosine levels, which were significantly higher in asthmatic children than those in healthy children [204].

### The role of BD in smoking-associated diseases

Tobacco smoke contains over 6,000 compounds, of which more than 70 compounds have been classified as human carcinogens [205–207]. BD is one of the carcinogens with the largest amounts in mainstream smoke; its typical yield is 52 μg/cigarette, which is more than 400-fold higher than 4-(methylnitrosamino)-1-(3-pyridyl)-1-butanone (NNK), a strong tobacco-specific carcinogen [208]. In terms of cancer risk, BD has been ranked as the most carcinogenic compound in cigarette smoke among 40 carcinogens with cancer potency factors being available [209].

Tobacco smoking has been associated with an array of diseases, including many types of cancers (e.g., lung cancer, liver cancer, bladder cancer, and leukemia), diabetes mellitus, CVD, asthma, etc. [210]. In spite of the ranking of BD as the top carcinogen in cigarette smoke, the contribution of BD in tobacco smoking-associated diseases, in particular, cancers, has not been elucidated.

BD is listed as a likely but minor causative agent for cigarette smoke-related lung and larynx cancers, mostly based on its tumorigenesis in mice [205, 208, 211]. However, the latest epidemiological study on more than 20,000 workers employed at eight North American synthetic rubber polymer plants from 1943 through 2009, which is the largest cohort for occupational exposure to BD, found that there was no causal association between BD exposure and lung cancer [212]. This study and the authors’ earlier investigations indeed observed elevated rates of lung cancer among female workers, however, the authors thought that it may be caused by other factors rather than exposure to BD due to the lack of a positive exposure-response trend [172, 213–215]. The observation also obtains support from other independent studies, including an investigation on the relationships between several carcinogens in cigarette smoke and lung cancer in a Chinese cohort [216], an ecological study at the country level conducted in the United States [217], and an investigation on cancer incidence in a petrochemical industry area in Sweden [218]. Moreover, in a U.S. study to compare the BD metabolism in smokers in three ethnic/racial groups (Native Hawaiians, whites, and Japanese Americans), Park et al. found that the MHBMA levels in Native Hawaiian smokers were significantly lower compared to those in white smokers, suggesting that Native Hawaiian smokers had lower BD uptake rates or metabolized BD to EB and then MHBMA less efficiently than whites [219]. However, it has been established that for the same lifetime smoking exposure, Native Hawaiian smokers have a significantly greater risk of lung cancer than do whites [220]. Thus, the finding of Park et al. actually provides indirect support for the epidemiological observation that BD exposure has no causal association with lung cancer. Collectively, the studies suggest that BD is not a *human* lung carcinogen and thus is not expected to contribute to smoking-associated lung cancer.

In spite of the fact that BD is listed as a minor contributing agent for larynx cancer [205], in fact, the studies concerning BD exposure and larynx cancer have been very scarce. A few studies to investigate the mortality in workers from the styrene-BD rubber industry and the BD production industry found that certain subgroups of workers had more than expected deaths from larynx cancer [175, 176, 215]. However, the results were all based on small numbers and thus were statistically imprecise. Moreover, the excess in larynx cancer was not clearly associated with any process group, suggesting that the increases were not due to BD exposure [215]. It should be noted that a positive association has indeed been observed between occupational exposures in the rubber-manufacturing industry and larynx cancer [4]. However, the observation is unable to relate the development of larynx cancer to exposure to specific chemicals because the rubber-manufacturing industry uses a wide variety of substances. In addition, a review of literature on the contribution of various types of occupational exposure to rare cancers does not list BD as a possible contributor to larynx cancer [221].

Because cigarette smoke is a known risk factor for urothelial carcinoma, a recent case-control study investigated the relationships among smoking, urinary levels of several VOC biomarkers, and urothelial carcinoma risk. However, no association between the levels of MHBMA3 or DHBMA and the risk of urothelial carcinoma has been discovered [144].

Taken together, it appears that BD may not contribute to pathogenesis of cigarette smoking-associated cancers of lung and larynx. Because BD has been associated with leukemia and some types of childhood tumors, and non-carcinogenic effects such as CVD and asthma, it is likely that BD is involved in these diseases caused by smoking. However, to our knowledge, so far no studies to investigate the associations have been published.

## Discussion and perspective

The studies of BD over the last decade have brought some important developments, among which the adverse effects on children’s health are probably the most profound issue. However, more carefully-designed studies are needed to provide further evidence for these findings.

In epidemiological studies, it is a critical issue to determine human exposure to BD. Currently, most epidemiological studies estimated human exposure to BD based on its concentrations in the ambient air. However, the approach can cause great uncertainties for studies conducted in the general population. As pointed out by Fujita et al., estimates of population exposure to air pollutants extrapolated from ambient measurements at ambient fixed site monitors are prone to uncertainty [48]. Multiple studies have indicated that microenvironments, especially those at home, are the main determinant of human exposure and the BD concentrations in microenvironments are often unrelated to those in the ambient air. Moreover, several population studies has consistently demonstrated that with regard to human exposure to BD, ETS is the primary source in the general population. Therefore, for epidemiological studies on the general population, a reliable estimate of human exposure to BD is desirable. Currently, the best approach can be using the urinary levels of BD biomarkers (preferably MHBMA3) as the metric for assessing human exposure, although it should be kept in mind that the urinary biomarkers reflect only recent exposures.

The finding that BD has natural sources from trees and soil bacteria is intriguing. However, so far only two tree species and a strain of soil bacterium have been reported to release BD. Thus, it is worth further exploration whether other species of plants and bacteria can also generate BD. If the quantities generated by plants are large enough, it may have an impact on human exposure to BD and it is also likely that BD can play a role in atmosphere chemistry.

Because BD must undergo bioactivation to exert its toxic effects, including mutagenicity/carcinogenicity, obviously identifying the specific BD metabolite(s) that dominantly contribute to the toxic effects, particularly in humans, is at the core of the underlying molecular mechanisms of BD toxicity. However, so far the issue has not been addressed, largely because the metabolism of BD is quite complex and more than 10 potential metabolites, most of which are genotoxic, can be formed [1, 101, 102]. Making matters worse, each of the major metabolites, e.g., EB, DEB, EBD, and CHB, contains stereoisomers due to the presence of chiral carbon atoms in these molecules, and importantly, the stereoisomers can have different bioactivity (e.g., cytotoxicity and mutagenicity) [6, 7, 94, 99]. In addition, the metabolism of BD has great species difference [1, 6, 7], leading to extra difficulty for studies on humans. Nonetheless, addressing the issue should be a major research direction for future studies.

Currently, it is generally assumed that mutagenicity/carcinogenicity of BD is caused by the epoxide metabolites formed via the P450-mediated metabolism. Interestingly, all epidemiological studies that have been published so far have not discovered any associations between BD exposure and cancers of lung, liver, and kidney, the major organs that are responsible for uptake and biotransformation of BD. Counterintuitively, the target organ for BD carcinogenicity in humans is the lymphohematopoietic system, an organ that seems not to be involved in the formation of P450-mediated epoxides. Why BD is not carcinogenic in the major human organs for uptake and metabolism is an intriguing issue. While extrahepatic toxicity is a likely explanation, the MPO-mediated metabolic pathway seems to provide an attractive alternate because the pathway is expected to occur exactly in the lymphohematopoietic system. Addressing the issue is an important research direction because it concerns the molecular mechanisms of BD mutagenicity/carcinogenicity and can have significant impact on the risk assessment of BD.

Although having been studied for 40 years, our understanding of the molecular mechanisms of BD toxicity has still been limited. Besides the associations with diseases that have been discovered, it is likely that BD contributes to other adverse effects on human health or the etiology of other diseases, in particular, as a factor to promote disease progression rather than as a disease-initiating factor. Considering that BD is a ubiquitous pollutant, the direction deserves more attention.

## Conclusions

BD is a ubiquitous environmental mutagen/carcinogen with high cancer risk. Its major environmental sources include automobile exhaust and tobacco smoke. The studies over the last decade have shown that microenvironments, particularly indoor microenvironments, are the primary determinant of exposure of the general population to BD, with tobacco smoke being the main source. While it has been known for more than 20 years that occupational exposure to BD is associated with leukemia and CVD, recent progress shows that non-occupational exposure to BD may be associated with certain reproductive effects, and more importantly, with several childhood cancers, autism, and asthma. The adverse effects on children’s health need special attention and more studies.

## Data Availability

Not applicable.

## Abbreviations

ADH: alcohol dehydrogenase
ALL: acute lymphocytic leukemia
AML: acute myeloid leukemia
BD: 1,3-butadiene
BDD: 3-butene-1,2-diol
*bis*-BDMA: 1,4-*bis*(*N*-acetyl-L-cystein-*S*-yl)butane-2,3-diol
*bis*-N7G-BD: 1,4-*bis*(gua-7-yl)-2,3-butanediol
CBMN: cytokinesis-block micronucleus
CBO: 1-chloro-2-buten-2-one
CEB: 1-chloro-3,4-epoxy-2-butanol
CHB: 1-chloro-2-hydroxy-3-butene
CI: confidence interval
CPD: cigarettes smoked per day
CVD: cardiovascular disease
DEB: 1,2,3,4-diepoxybutane
DHBMA: *N*-acetyl-*S*-(3,4-dihydroxybutyl)-L-cysteine
EB: 3,4-epoxy-1-butene
EB-GI: *N*7-(2-hydroxy-3-buten-1-yl)guanine
EB-GII: *N*7-[1-(hydroxymethyl)-2-propen-1-yl]guanine
EBD: 3,4-epoxybutane-1,2-diol
EPA: the U.S. Environmental Protection Agency
ETS: environmental tobacco smoke
GCTs: germ cell tumors
GSH: glutathione
HAPs: hazardous air pollutants
HMVK: hydroxymethyl vinyl ketone
HPRT: hypoxanthine-guanine phosphoribosyltransferase
IARC: the International Agency for Research on Cancer
IQR: interquartile range
LOD: limit of detection
MHBMA: monohydroxybutenyl mercapturic acid
MHBMA1: *N*-acetyl-*S*-[1-(hydroxymethyl)-2-propen-1-yl]-L-cysteine
MHBMA2: *N*-acetyl-*S*-(2-hydroxy-3-buten-1-yl)-L-cysteine
MHBMA3: *N*-acetyl-*S*-(4-hydroxy-2-buten-1-yl)-L-cysteine
MN: micronucleus
MPO: myeloperoxidase
NAC: *N*-acetyl-L-cysteine
NC1: 1,4-*bis*(*N*-acetyl-L-cystein-*S*-yl)-2-butanone
NNK: 4-(methylnitrosamino)-1-(3-pyridyl)-1-butanone
NPB: nucleoplasmic bridge
OR: odds ratio
P450s: cytochrome P450 enzymes
PBL: peripheral blood lymphocytes
PNET: primitive neuroectodermal tumor
RR: rate ratio
SCE: sister-chromatid exchange
SMR: standardized mortality ratio
TBW: term birth weight
THBMA: *N*-acetyl-*S*-(2,3,4-trihydroxybutyl)-L-cysteine
VOCs: volatile organic compounds
